# RET Proto-Oncogene Variants in Patients with Medullary Thyroid Carcinoma from the Mediterranean Basin: A Brief Report

**DOI:** 10.3390/life13061332

**Published:** 2023-06-06

**Authors:** Vassos Neocleous, Pavlos Fanis, Savvas Frangos, Nicos Skordis, Leonidas A. Phylactou

**Affiliations:** 1 Department of Molecular Genetics, Function and Therapy, The Cyprus Institute of Neurology and Genetics, Nicosia 2371, Cyprus; vassosn@cing.ac.cy (V.N.); pavlosf@cing.ac.cy (P.F.); nicosskordis@paedi.org.cy (N.S.); 2 Nuclear Medicine Department, Bank of Cyprus Oncology Center, Nicosia 2404, Cyprus; savvas.frangos@gmail.com; 3 Division of Paediatric Endocrinology, Paedi Center for Specialized Paediatrics, Nicosia 2024, Cyprus; 4 School of Medicine, University of Nicosia, Nicosia 2417, Cyprus

**Keywords:** *RET* proto-oncogene, MEN2, medullary thyroid carcinoma, Mediterranean Basin

## Abstract

Multiple endocrine neoplasia type 2 (MEN2) is an autosomal dominant (AD) condition with very high penetrance and expressivity. It is characterized into three clinical entities recognized as MEN2A, MEN2B, and familial medullary thyroid carcinoma (FMTC). In both MEN2A and MEN2B, there is a manifestation of multicentric tumor formation in the major organs such as the thyroid, parathyroid, and adrenal glands where the *RET* proto-oncogene is expressed. The FMTC form differs from MEN2A and MEN2B, since medullary thyroid carcinoma (MTC) is the only feature observed. In this present brief report, we demonstrate a collection of *RET* proto-oncogene genotype data from countries around the Mediterranean Basin with variable characteristics. As expected, a great extent of the Mediterranean *RET* proto-oncogene genotype data resemble the data reported globally. Most interestingly, higher frequencies are observed in the Mediterranean region for specific pathogenic *RET* variants as a result of local prevalence. The latter can be explained by founder effect phenomena. The Mediterranean epidemiological data that are presented herein are very important for domestic patients, their family members’ evaluation, and ultimately their treatment.

## 1. Introduction

The prevalence of multiple endocrine neoplasia 2 (MEN2) which is subdivided into MEN2A and MEN2B is estimated to account for 1–3% of all thyroid malignant tumors [1,2]. MEN2A is an inherited disorder and is associated with three main types of malignancies: MTC in 95%, pheochromocytoma in 50%, and parathyroid adenoma in 5–10% of the incidences [2]. MEN2B manifests the most rare and aggressive form of MEN2 and is associated with MTC, pheochromocytoma, marfanoid habitus, and mucosal and intestinal ganglioneuromatosis [3,4].

Both MEN2A and MEN2B are caused by *RET* gene germline pathogenic variants [2,5,6]. The *RET* gene encodes a tyrosine kinase glycoprotein receptor that plays a role in the transduction of signals during proliferation and differentiation stages in the neural crest. The first *RET* pathogenic variants were reported in 1993 by two independent groups [7,8], and since then, more than 100 pathogenic *RET* variants have also been identified in a large percentage of MEN2A patients who were clinically diagnosed with MTC, pheochromocytoma, or both [6,9,10]. The most frequent pathogenic variants are positioned in exons 10, 11, 13, 14, 15, and 16 of the *RET* gene, and according to the American Thyroid Association (ATA) and the European Thyroid Association (ETA), they are classified into three levels: highest (ATA-HST), high (ATA-H), and moderate risk (ATA-MOD) [2,10,11,12,13]. Concomitantly, several of those classified as MEN2 causing germline pathogenic variants in exon 10 of the *RET* gene in codons 609, 611, 618, and 620 have also been reported in Hirschsprung disease [2,5]. Several studies have shown that as a result of pathogenic variants in codons 609, 618, and 620, malignant pheochromocytomas can be determined as belligerent as compared to the ones caused by the exon 11 five amino acid variations of codon 634 [10,14,15]. Nevertheless, the majority of the cases exhibited the highest countenance for 634, followed by a diminishing manner for codons 609, 618, and 620 [16,17,18,19]. Recent geographical reports regarding the spectrum of pathogenic variants reported in the *RET* gene in individuals with MEN2 have also brought to light the clinically less aggressive forms of the disease, as a result of mutations in exons 13–15 of the gene [1,20,21,22]. 

The objective of this brief review article is to produce a summary of the existing *RET* pathogenic variants and their phenotypes in countries from the Mediterranean Basin. The heterogeneity of pathogenic *RET* variants that is observed globally rationalizes the need to further define the epidemiological data and genetic background from a historical and exclusive region, such as the Mediterranean Basin.

### 1.1. Geographical Distribution of RET Pathogenic Variants in the Mediterranean Region

The Mediterranean region has always been a place of migratory flows. During the European Neolithic expansion period (7000–2000 BCE), genetic influxes along with new stone practices for making tools were introduced into endemic populations as a result of groups of people with specific genetic material [23,24]. 

#### 1.1.1. *RET* Pathogenic Variants in Italy

**Figure 1:**
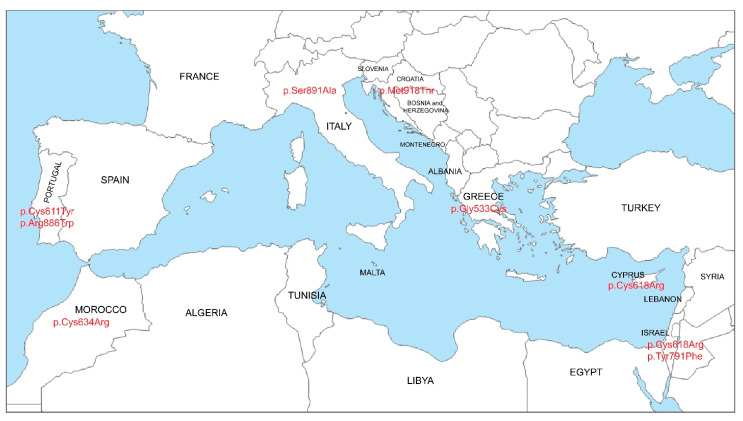
High frequency of the founder mutations that are most commonly seen in seven different countries around the Mediterranean Basin.

To date, the genetic makeup of Italian patients with MEN2 is quite comprehensive. According to ATA guidelines, the pathogenic variants at codon 634 occupy about 36% of the reported *RET* pathogenic variants, followed by the two moderate risk variants p.Val804Met (21.9%) and p.Ser891Ala (9.7%) [2,10,21,25,26] (Table 1). The high frequency of p.Val804Met was first described in the Sardinian population with a 59% prevalence which was initially attributed to the role of genetic drift and the effect phenomenon [26]. A few years later, haplotype analysis for p.Val804 excluded the possibility of a founder effect in Sardinia and the Italian peninsula [1] (Table 1). An estimated 6.3% of the Italian patients were also genotyped with the ATA-MOD risk *RET* pathogenic variants at codon 618 in exon 10 [10] (Table 1). A recent report covering patients with MTC from Northern Italy identified an unexpectedly high frequency of the ATA-MOD risk p.Ser891Ala and uncovered a founder effect phenomenon [27] (Figure 1). More specifically, all of the 28 unrelated patients from Northern Italy shared a common haplotype, estimating that they all had a common ancestor dating back to 1493 AD [27]. The MEN2B phenotype, caused by the ATA-HST risk *RET* pathogenic p.Met918Thr variant, was likewise found in patients of Italian descent and its prevalence (6.8%) corresponded to between 4.6–13.5% of the affected patients [1,10] (Table 1). 

#### 1.1.2. *RET* Pathogenic Variants on the Iberian Peninsula: Spain and Portugal

In Spain, numerous studies over the last two decades have demonstrated the ATA-H risk *RET* pathogenic variant p.Cys634Tyr to be the most frequent in nearly 70% of MEN2A cases, followed by the ATA-HST risk p.Cys634Arg, which is the predominant variant in the rest of Europe [28,29,30,31,32,33] (Table 1). Reports from various countries with high prevalence of p.Cys634Tyr and p.Cys634Arg have confirmed that the clinical phenotype of the latter usually confers a more aggressive MEN2A phenotype [14,32]. Other less frequent germline missense pathogenic variants of variable expressivity have also been detected in Spanish patients with MEN2A in codons 609, 611, 618, 620, and 630 [10,28,29] (Table 1). The ATA-HST pathogenic p.Met918thr variant was also identified in Spanish patients with MEN2B, and its prevalence ranged between 4.6–13.5% of the affected patients [10] (Table 1). 

Since they are both located on the Iberian Peninsula and the fact that Spain and Portugal are two of the oldest countries in Europe, one would expect their genetic makeup to be similar. This is not the case though based on the identified *RET*-proro-oncogene pathogenic variants reported so far. In the recent comprehensive review by Maciel et al. [10], only 3 out of 20 (p.Cys634Arg, p.Cys634Tyr, and p.Met918Thr) reported *RET* proto-oncogene pathogenic variants are common between Spanish and Portuguese patients [27,34,35,36,37,38,39,40,41] (Table 1). In Portugal, a total of fourteen *RET* pathogenic variants have so far been reported. Interestingly, two of these pathogenic variants, p.Cys611Tyr and p.Arg886Trp, were found in cases from the central region of Portugal and they all shared a common haplotype, thus suggesting a possible founder effect [10,34,41] (Table 1).

#### 1.1.3. *RET* Pathogenic Variants in France 

There are currently no specific reports regarding the prevalence of *RET* pathogenic variants that are exclusively found in the Southern part of France and the major metropolitan cities located close to the Mediterranean Sea. The current French data regarding the *RET* pathogenic variants emanate from past and recent reports, such as the multicenter study directed by the French Group of Endocrine Tumors which included information from 18 different centers and a collection of others [42,43,44,45]. As depicted in the recent geographical variation review by Maciel et al. [10], the most frequent *RET* pathogenic variants in a large French cohort of MEN2 patients (*n* = 437) were the ATA-H risk *RET* pathogenic variant p.Cys634(Phe/Gly/Arg/Ser/Trp/Tyr) (32.9%), followed by the ATA-MOD risk p.Val804(Leu/Met) (21.0%) and p.Leu790Phe (9.9%) (Table 1). The ATA-H risk p.Cys634Arg, which is the predominant variant in the rest of Europe, was also found in 6.9% of the patients from this specific French cohort [10,42,43] (Table 1).

#### 1.1.4. *RET* Pathogenic Variants in Greece and Cyprus

The present territory of modern Greece as a Mediterranean entity was unavoidably influenced during the Neolithic expansion era by the arrival of immigrant farmers from the Near East [46]. During this distinctive period, along with the obvious innovative stone-age technologies that were brought to the indigenous population, new genomic variations were also introduced, as a result of multi-phase mixing contributing in that way to their genetic distinctiveness [46,47]. 

In Greek patients with MEN2, a total of twelve pathogenic variants have so far been reported, with the MEN2A causing ATA-MOD risk p.Gly533Cys (36.3%) and ATA-MOD risk p.Cys618Arg/Tyr/Ser (32.8%) being the most frequent types, followed by the MEN2A causing ATA-MOD risk p.Cys620Tyr/Arg/Phe, the ATA-H risk p.Cys634Arg/Tyr/Phe, and the ATA-MOD risk p.Leu804Met and the MEN2B causing ATA-HST risk p.Met918Thr [10,48,49,50,51] (Table 1). 

Interestingly, several studies that have investigated MEN2A patients from Greece reported the ATA-MOD risk p.Gly533Cys to be the most prevalent pathogenic variant and that it resulted from a possible founder effect phenomenon [48,49,50,51,52,53,54] (Figure 1). Not long ago, the p.Gly533Cys pathogenic variant was also found in four patients from a Brazilian cohort, and the possibility of a common ancestral origin was examined along with another unrelated eight carriers of Greek descent. Not unexpectedly, all p.Gly533Cys carriers shared an identical core haplotype, indicating a common ancestor, and as a result of the frequent migratory currents in the Mediterranean region, this was also likely taken to the Iberian Peninsula [53,55,56]. Recent reports from Cyprus and Israel identified the ATA-MOD risk p.Cys618Arg variant as the most prevalent, found in 69% and 55% of the probands, respectively [57,58,59,60,61]. The p.Cys618Arg variant was reported in nine apparently unrelated families from Cyprus who all shared a common haplotype (Table 1). This finding was explained as an ancestral pathogenic variant which prevailed on the island as a result of a possible founder effect [57] (Figure 1). 

#### 1.1.5. MEN2-Related *RET* Pathogenic Variants from Other Mediterranean Countries

The prevalent p.Cys618Arg variant in Israel in nearly all Israeli cases that have been reported so far is linked to two large families of Moroccan Jewish descent with familial medullary thyroid carcinoma and Hirschsprung disease [62] (Table 1). As a result of the close proximity of Israel to Cyprus, the possibility of a common ancestor and a shared haplotype cannot be excluded and could be the subject of an investigation in a future study. Interestingly, the p.Tyr791Phe *RET* variant that has been considered as a putative Slavic ancestry variant because of its high prevalence in the Czech Republic, Poland, and Central Europe has also been frequently reported in sporadic appearing and less aggressive MTC cases in Ashkenazi Jews, thus also implying a possible founder mutation [63] (Figure 1). A recent review detected that few reports have pointed to the misclassification of *RET* p.Tyr791Phe as a likely pathogenic variant and, therefore, to the manifestation of needless thyroidectomies. The present notion is that the *RET* p.Tyr791Phe alone shows no association with MTC susceptibility and only when inherited *in cis* with p.Cys634Tyr does it confer penetrance of MTC and pheochromocytoma [64,65]. 

In a retrospective multicenter study, it was demonstrated that patients with MTC from Turkey had a comparable pathogenic *RET* variant distribution when compared to other Mediterranean countries including Italy and France [66]. Among the reported patients identified with *RET* mutations, p.Cys634Arg was the most prevalent (54.9%), followed by p.Val804Met (25.4%) and p.Cys634Tyr (8.5%) [66,67,68] (Table 1). 

Several reports from Slovenia, a Mediterranean country with a border in the Adriatic Sea, determined the type of *RET* mutation in Slovenian MTC patients [69,70]. A recent report from the Cancer Registry of the Republic of Slovenia specified that the basic yearly incidence rate of MTC in the Slovenian population was 0.34/100,000, as 143 patients were identified with MTC between 1995 and 2015 [71,72]. The most frequently altered codons in MTC patients from Slovenia were not different from those reported in the other Mediterranean neighboring countries, such as France and Italy, and were mainly the codons 634 and 618, followed by codon 790, codon 804, and codon 918 [69,70,71,72] (Table 1). 

In the neighboring country of Croatia with borders also in the Adriatic Sea, a recent report described a cohort of 21 MTC patients all sharing MEN2B causing ATA-HST risk p.Met918Thr which seems to be quite frequent in this generally isolated population [73] (Table 1). 

Since Morocco is a neighboring country located in the northwest corner of Africa and also bordered by the Mediterranean Sea, it is somewhat anticipated that the *RET* pathogenic variant spectra detected in Moroccan patients with MEN2 are comparable to those previously described in other nations. Up-to-date Moroccan MTC patients have only been reported with three ATA-H risk germline mutations in codon 634 (p.Cys634Arg/Phe/Trp), and this finding could be elucidated as an outcome of genetic drift or a founder effect phenomenon regarding the history of these people who lived remote for numerous hundreds of years [74,75] (Figure 1).

**Table 1:** Mutations and founder effect phenomena described in countries of the Mediterranean region with their corresponding clinical phenotype.

Mediterranean Country	ATA Category/ Clinical Phenotype	RET Protein Change	Founder Effect
Italy	ATA-H/MEN2A ATA-MOD/MEN2A ATA-MOD/MEN2A ATA-HST/MEN2B	p.Cys634Arg/Gly/Phe/Ser/Trp/Tyr/Val [10,21,25]; p.Cys618Arg/Tyr/Gly [10,21,25]; p.Val804Met [26]; p.Ser891Ala [27] p.Met918Thr [10,21]	p.Ser891Ala (Northern Italy) [27]
Spain	ATA-H/MEN2A ATA-MOD/MEN2A ATA-MOD/MEN2A ATA-HST/MEN2B	p.Cys634Tyr/Arg [10,28,29,30,31,32,33]; p.Cys609Ser, p.Cys611Phe, p.Cys618Arg, p.Cys620Ser, p.Ser589Cys, p.Glu768Asp, p.Val804Met, p.Ser891Ala [10,29] p.Met918Thr [10,29,30]	
Portugal	ATA-H/MEN2A ATA-MOD/MEN2A ATA-MOD/MEN2A ATA-MOD/MEN2A ATA-MOD/MEN2A ATA-HST/MEN2B	p.Cys634Tyr/Arg, p.Ala883Phe [35,36,37]; p.Cys515Trp, p.Cys531Arg, p.Cys609Arg, p.Cys611Tyr, p.Cys620Arg, p.Cys630Gly, p.Thr636Met, p.Ser649Leu, p.Val804Leu, p.Arg886Trp [34,36,38,39,41]; p.Met918Thr [36,37]	p.Cys611Tyr (Central Portugal) [34]; p.Arg886Trp (Central Portugal) [34]
France	ATA-H/MEN2A ATA-MOD/MEN2A ATA-MOD/MEN2A ATA-HST/MEN2B	p.Cys634(Phe/Gly/Arg/Ser/Trp/Tyr) [42,43]; p.Cys618Phe/Gly/Arg/Ser/Tyr [42,43]; p.Leu790Phe, p.Val804(Leu/Met) [42,43]; p.Met918Thr [43]	
Greece	ATA-H/MEN2A ATA-MOD/MEN2A ATA-MOD/MEN2A ATA-HST/MEN2B	p.Cys634Arg/Tyr/Phe [51]; p.Gly533Cys, p.Cys618Arg/Tyr/Ser; p.Cys620Tyr/Arg/Phe, p.Leu804Met [49,51,52,53,54]; p.Met918Thr [51,76]	p.Gly533Cys (Central-Western Greece) [52,53,54]
Israel	ATA-H/MEN2A ATA-MOD/MEN2A ATA-HST/MEN2B	p.Cys634Arg [59,60]; p.Cys618Arg, p.Val804Met, p.Tyr791Phe [62,63] *; p.Met918Thr [60]	p.Cys618Arg (Moroccan Jewish descent) [62] *; p.Tyr791Phe (Ashkenazi Jews) [63]
Cyprus	ATA-MOD/MEN2A ATA-HST/MEN2B	p.Cys618Arg, p.Glu632_Leu633del [18,57,58]; p.Met918Thr [57]	p.Cys618Arg (Southern Cyprus) [57,58]
Turkey	ATA-H/MEN2A ATA-MOD/MEN2A	p.Cys634Arg/Tyr [66,67]; p.Val804Met [68]	
Slovenia	ATA-H/MEN2A ATA-MOD/MEN2A ATA-HST/MEN2B	p.Cys634Arg/Tyr/Gly [69,70,71,72]; p.Cys618Arg/Ser, p.Leu790Phe, p.Val804Met, p.Ala639Thr [69,70,71,72] p.Met918Thr [69,70,71,72]	
Croatia	ATA-HST/MEN2B	p.Met918Thr **	p.Met918Thr (Isolated population possible founder effect) **
Morocco	ATA-H/MEN2A	p.Cys634Arg/Phe/Trp [74,75]	p.Cys634Arg/Phe/Trp (Isolated population possible founder effect) [74,75]

ATA: American Thyroid Association, ATA-HST: highest risk, ATA-H: high risk, and ATA-MOD: moderate risk. * Moroccan Jewish descent; ** Isolated population possible founder effect.

## 2. Conclusions

The region enclosing the Mediterranean Sea is a densely populated area and is historically characterized by complicated and intense human activity between the various ethnic groups. Inevitably, this irregularity has also led to genetic heterogeneity of various inherited diseases including MEN2. In the current review, we present the phenotypic spectrum of *RET* pathogenic variants reported so far that have been transmitted to offspring for centuries. Interestingly, several haplotype analysis studies delineated the high prevalence of specific *RET* pathogenic variants in specific ethnic groups to be the result of founder effect phenomena. In the years to come, the use of contemporary diagnostic approaches, such as next-generation sequencing, will enable more countries from around the globe including the Mediterranean Basin to be analyzed for more and explicit diagnoses regarding MEN2. 

## Data Availability

Not applicable.

## References

[1] Romei C., Mariotti S., Fugazzola L., Taccaliti A., Pacini F., Opocher G., Mian C., Castellano M., Degli Uberti E., Ceccherini I. (2010). Multiple endocrine neoplasia type 2 syndromes (MEN 2): Results from the ItaMEN network analysis on the prevalence of different genotypes and phenotypes. Eur. J. Endocrinol..

[2] Wells S.A., Asa S.L., Dralle H., Elisei R., Evans D.B., Gagel R.F., Lee N., Machens A., Moley J.F., Pacini F. (2015). Revised American Thyroid Association guidelines for the management of medullary thyroid carcinoma. Thyroid.

[3] Castinetti F., Moley J., Mulligan L., Waguespack S.G. (2018). A comprehensive review on MEN2B. Endocr. Relat. Cancer.

[4] Znaczko A., Donnelly D.E., Morrison P.J. (2014). Epidemiology, clinical features, and genetics of multiple endocrine neoplasia type 2B in a complete population. Oncologist.

[5] Eng C., Clayton D., Schuffenecker I., Lenoir G., Cote G., Gagel R.F., van Amstel H.K., Lips C.J., Nishisho I., Takai S.I. (1996). The relationship between specific RET proto-oncogene mutations and disease phenotype in multiple endocrine neoplasia type 2. International RET mutation consortium analysis. JAMA.

[6] Raue F., Bruckner T., Frank-Raue K. (2019). Long-Term Outcomes and Aggressiveness of Hereditary Medullary Thyroid Carcinoma: 40 Years of Experience at One Center. J. Clin. Endocrinol. Metab..

[7] Mulligan L.M., Kwok J.B., Healey C.S., Elsdon M.J., Eng C., Gardner E., Love D.R., Mole S.E., Moore J.K., Papi L. (1993). Germ-line mutations of the RET proto-oncogene in multiple endocrine neoplasia type 2A. Nature.

[8] Donis-Keller H., Dou S., Chi D., Carlson K.M., Toshima K., Lairmore T.C., Howe J.R., Moley J.F., Goodfellow P., Wells S.A. (1993). Mutations in the RET proto-oncogene are associated with MEN 2A and FMTC. Hum. Mol. Genet..

[9] Mulligan L.M. (2014). RET revisited: Expanding the oncogenic portfolio. Nat. Rev. Cancer.

[10] Maciel R.M.B., Maia A.L. (2021). Global Endocrinology: Geographical variation in the profile of RET variants in patients with medullary thyroid cancer: A comprehensive review. Eur. J. Endocrinol..

[11] Kloos R.T., Eng C., Evans D.B., Francis G.L., Gagel R.F., Gharib H., Moley J.F., Pacini F., Ringel M.D., Schlumberger M. (2009). Medullary thyroid cancer: Management guidelines of the American Thyroid Association. Thyroid.

[12] Elisei R., Alevizaki M., Conte-Devolx B., Frank-Raue K., Leite V., Williams G.R. (2013). 2012 European thyroid association guidelines for genetic testing and its clinical consequences in medullary thyroid cancer. Eur. Thyroid J..

[13] Brandi M.L., Gagel R.F., Angeli A., Bilezikian J.P., Beck-Peccoz P., Bordi C., Conte-Devolx B., Falchetti A., Gheri R.G., Libroia A. (2001). Guidelines for diagnosis and therapy of MEN type 1 and type 2. J. Clin. Endocrinol. Metab..

[14] Punales M.K., Graf H., Gross J.L., Maia A.L. (2003). RET codon 634 mutations in multiple endocrine neoplasia type 2: Variable clinical features and clinical outcome. J. Clin. Endocrinol. Metab..

[15] Milos I.N., Frank-Raue K., Wohllk N., Maia A.L., Pusiol E., Patocs A., Robledo M., Biarnes J., Barontini M., Links T.P. (2008). Age-related neoplastic risk profiles and penetrance estimations in multiple endocrine neoplasia type 2A caused by germ line RET Cys634Trp (TGC>TGG) mutation. Endocr. Relat. Cancer.

[16] Lindskog S., Nilsson O., Jansson S., Nilsson B., Illerskog A.C., Ysander L., Ahlman H., Tisell L.E. (2004). Phenotypic expression of a family with multiple endocrine neoplasia type 2A due to a RET mutation at codon 618. Br. J. Surg..

[17] Quayle F.J., Fialkowski E.A., Benveniste R., Moley J.F. (2007). Pheochromocytoma penetrance varies by RET mutation in MEN 2A. Surgery.

[18] Neocleous V., Passalaris T., Spanou E., Kitsios P., Skordis N., Deltas C.C. (2004). Description of the first two seemingly unrelated Greek Cypriot families with a common C618R RET proto-oncogene mutation. Genet. Test..

[19] Mucha L., Leidig-Bruckner G., Frank-Raue K., Bruckner T., Kroiss M., Raue F., German Study Group for Rare Thyroid Cancer (2017). Phaeochromocytoma in multiple endocrine neoplasia type 2: RET codon-specific penetrance and changes in management during the last four decades. Clin. Endocrinol..

[20] Machens A., Lorenz K., Sekulla C., Hoppner W., Frank-Raue K., Raue F., Dralle H. (2013). Molecular epidemiology of multiple endocrine neoplasia 2: Implications for RET screening in the new millenium. Eur. J. Endocrinol..

[21] Romei C., Tacito A., Molinaro E., Agate L., Bottici V., Viola D., Matrone A., Biagini A., Casella F., Ciampi R. (2015). Twenty years of lesson learning: How does the RET genetic screening test impact the clinical management of medullary thyroid cancer?. Clin. Endocrinol..

[22] Saravana-Bawan B., Pasternak J.D. (2022). Multiple endocrine neoplasia 2: An overview. Ther. Adv. Chronic. Dis..

[23] Childebayeva A., Rohrlach A.B., Barquera R., Rivollat M., Aron F., Szolek A., Kohlbacher O., Nicklisch N., Alt K.W., Gronenborn D. (2022). Population Genetics and Signatures of Selection in Early Neolithic European Farmers. Mol. Biol. Evol..

[24] Mathieson I., Lazaridis I., Rohland N., Mallick S., Patterson N., Roodenberg S.A., Harney E., Stewardson K., Fernandes D., Novak M. (2015). Genome-wide patterns of selection in 230 ancient Eurasians. Nature.

[25] Elisei R., Tacito A., Ramone T., Ciampi R., Bottici V., Cappagli V., Viola D., Matrone A., Lorusso L., Valerio L. (2019). Twenty-Five Years Experience on RET Genetic Screening on Hereditary MTC: An Update on The Prevalence of Germline RET Mutations. Genes.

[26] Pinna G., Orgiana G., Riola A., Ghiani M., Lai M.L., Carcassi C., Mariotti S. (2007). RET proto-oncogene in Sardinia: V804M is the most frequent mutation and may be associated with FMTC/MEN-2A phenotype. Thyroid.

[27] Giacche M., Panarotto A., Tacchetti M.C., Tosini R., Campana F., Mori L., Cappelli C., Pirola I., Lombardi D., Pezzola D.C. (2019). p.Ser891Ala RET gene mutations in medullary thyroid cancer: Phenotypical and genealogical characterization of 28 apparently unrelated kindreds and founder effect uncovering in Northern Italy. Hum. Mutat..

[28] Sanchez B., Robledo M., Biarnes J., Saez M.E., Volpini V., Benitez J., Navarro E., Ruiz A., Antinolo G., Borrego S. (1999). High prevalence of the C634Y mutation in the RET proto-oncogene in MEN 2A families in Spain. J. Med. Genet..

[29] Fernandez R.M., Navarro E., Antinolo G., Ruiz-Ferrer M., Borrego S. (2006). Evaluation of the role of RET polymorphisms/haplotypes as modifier loci for MEN 2, and analysis of the correlation with the type of RET mutation in a series of Spanish patients. Int. J. Mol. Med..

[30] Rodriguez J.M., Balsalobre M., Ponce J.L., Rios A., Torregrosa N.M., Tebar J., Parrilla P. (2008). Pheochromocytoma in MEN 2A syndrome. Study of 54 patients. World J. Surg..

[31] Pomares F.J., Canas R., Rodriguez J.M., Hernandez A.M., Parrilla P., Tebar F.J. (1998). Differences between sporadic and multiple endocrine neoplasia type 2A phaeochromocytoma. Clin. Endocrinol..

[32] Valdes N., Navarro E., Mesa J., Casteras A., Alcazar V., Lamas C., Tebar J., Castano L., Gaztambide S., Forga L. (2015). RET Cys634Arg mutation confers a more aggressive multiple endocrine neoplasia type 2A phenotype than Cys634Tyr mutation. Eur. J. Endocrinol..

[33] Febrero B., Rodriguez J.M., Rios A., Segura P., Perez-Sanchez B., Torregrosa N., Hernandez A.M., Parrilla P. (2019). Prophylactic thyroidectomy in multiple endocrine neoplasia 2 (MEN2) patients with the C634Y mutation: A long-term follow-up in a large single-center cohort. Eur. J. Surg. Oncol..

[34] Prazeres H.J., Rodrigues F., Figueiredo P., Naidenov P., Soares P., Bugalho M.J., Lacerda M., Campos B., Martins T.C. (2006). Occurrence of the Cys611Tyr mutation and a novel Arg886Trp substitution in the RET proto-oncogene in multiple endocrine neoplasia type 2 families and sporadic medullary thyroid carcinoma cases originating from the central region of Portugal. Clin. Endocrinol..

[35] Bugalho M.J., Domingues R., Sobrinho L. (2003). MEN 2A families: From hot spots to hot regions. Int. J. Mol. Med..

[36] Bugalho M.J., Domingues R., Santos J.R., Catarino A.L., Sobrinho L. (2007). Mutation analysis of the RET proto-oncogene and early thyroidectomy: Results of a Portuguese cancer centre. Surgery.

[37] Moura M.M., Cabrera R.A., Esteves S., Cavaco B.M., Soares P., Leite V. (2021). Correlation of molecular data with histopathological and clinical features in a series of 66 patients with medullary thyroid carcinoma. J. Endocrinol. Investig..

[38] Martins A.F., Martins J.M., do Vale S., Dias T., Silveira C., da Silva I.R., Carmo-Fonseca M. (2016). A rare missense variant in RET exon 8 in a Portuguese family with atypical multiple endocrine neoplasia type 2A. Hormones.

[39] Prazeres H., Couto J.P., Rodrigues F., Vinagre J., Torres J., Trovisco V., Martins T.C., Sobrinho-Simoes M., Soares P. (2011). In vitro transforming potential, intracellular signaling properties, and sensitivity to a kinase inhibitor (sorafenib) of RET proto-oncogene variants Glu511Lys, Ser649Leu, and Arg886Trp. Endocr. Relat. Cancer.

[40] Silva A.L., Carmo F., Moura M.M., Domingues R., Espadinha C., Leite V., Cavaco B., Bugalho M.J. (2015). Identification and characterization of two novel germline RET variants associated with medullary thyroid carcinoma. Endocrine.

[41] Moura M.M., Cavaco B.M., Pinto A.E., Domingues R., Santos J.R., Cid M.O., Bugalho M.J., Leite V. (2009). Correlation of RET somatic mutations with clinicopathological features in sporadic medullary thyroid carcinomas. Br. J. Cancer.

[42] Rohmer V., Vidal-Trecan G., Bourdelot A., Niccoli P., Murat A., Wemeau J.L., Borson-Chazot F., Schvartz C., Tabarin A., Chabre O. (2011). Prognostic factors of disease-free survival after thyroidectomy in 170 young patients with a RET germline mutation: A multicenter study of the Groupe Francais d’Etude des Tumeurs Endocrines. J. Clin. Endocrinol. Metab..

[43] Lebeault M., Pinson S., Guillaud-Bataille M., Gimenez-Roqueplo A.P., Carrie A., Barbu V., Pigny P., Bezieau S., Rey J.M., Delvincourt C. (2017). Nationwide French Study of RET Variants Detected from 2003 to 2013 Suggests a Possible Influence of Polymorphisms as Modifiers. Thyroid.

[44] Veyrat-Durebex C., Bouzamondo N., Le Mao M., Chao de la Barca J.M., Bris C., Dieu X., Simard G., Gadras C., Tessier L., Drui D. (2019). Metabolomics signatures of a subset of RET variants according to their oncogenic risk level. Endocr. Relat. Cancer.

[45] Amodru V., Taieb D., Guerin C., Romanet P., Paladino N., Brue T., Cuny T., Barlier A., Sebag F., Castinetti F. (2020). Correction to: MEN2-related pheochromocytoma: Current state of knowledge, specific characteristics in MEN2B, and perspectives. Endocrine.

[46] Marchi N., Winkelbach L., Schulz I., Brami M., Hofmanova Z., Blocher J., Reyna-Blanco C.S., Diekmann Y., Thiery A., Kapopoulou A. (2022). The genomic origins of the world’s first farmers. Cell.

[47] Silva N.M., Kreutzer S., Souleles A., Triantaphyllou S., Kotsakis K., Urem-Kotsou D., Halstead P., Efstratiou N., Kotsos S., Karamitrou-Mentessidi G. (2022). Ancient mitochondrial diversity reveals population homogeneity in Neolithic Greece and identifies population dynamics along the Danubian expansion axis. Sci. Rep..

[48] Bethanis S., Koutsodontis G., Palouka T., Avgoustis C., Yannoukakos D., Bei T., Papadopoulos S., Linos D., Tsagarakis S. (2007). A newly detected mutation of the RET protooncogene in exon 8 as a cause of multiple endocrine neoplasia type 2A. Hormones.

[49] Peppa M., Boutati E., Kamakari S., Pikounis V., Peros G., Panayiotides I.G., Economopoulos T., Raptis S.A., Hadjidakis D. (2008). Multiple endocrine neoplasia type 2A in two families with the familial medullary thyroid carcinoma associated G533C mutation of the RET proto-oncogene. Eur. J. Endocrinol..

[50] Sarika H.L., Papathoma A., Garofalaki M., Vasileiou V., Vlassopoulou B., Anastasiou E., Alevizaki M. (2012). High prevalence of exon 8 G533C mutation in apparently sporadic medullary thyroid carcinoma in Greece. Clin. Endocrinol..

[51] Sarika H.L., Papathoma A., Garofalaki M., Saltiki K., Pappa T., Pazaitou-Panayiotou K., Anastasiou E., Alevizaki M. (2015). Genetic screening of patients with medullary thyroid cancer in a referral center in Greece during the past two decades. Eur. J. Endocrinol..

[52] Kaldrymides P., Mytakidis N., Anagnostopoulos T., Vassiliou M., Tertipi A., Zahariou M., Rampias T., Koutsodontis G., Konstantopoulou I., Ladopoulou A. (2006). A rare RET gene exon 8 mutation is found in two Greek kindreds with familial medullary thyroid carcinoma: Implications for screening. Clin. Endocrinol..

[53] Cunha L.L., Lindsey S.C., Franca M.I.C., Sarika L., Papathoma A., Kunii I.S., Cerutti J.M., Dias-da-Silva M.R., Alevizaki M., Maciel R.M.B. (2017). Evidence for the founder effect of RET533 as the common Greek and Brazilian ancestor spreading multiple endocrine neoplasia 2A. Eur. J. Endocrinol..

[54] Saltiki K., Anagnostou E., Simeakis G., Kouki S., Angelopoulou A., Sarika L., Papathoma A., Alevizaki M. (2017). Familial MTC with RET exon 8 Gly533Cys mutation: Origin and prevalence of second malignancy. Endocr. Connect..

[55] Da Silva A.M., Maciel R.M., Da Silva M.R., Toledo S.R., De Carvalho M.B., Cerutti J.M. (2003). A novel germ-line point mutation in RET exon 8 (Gly(533)Cys) in a large kindred with familial medullary thyroid carcinoma. J. Clin. Endocrinol. Metab..

[56] Paschou P., Drineas P., Yannaki E., Razou A., Kanaki K., Tsetsos F., Padmanabhuni S.S., Michalodimitrakis M., Renda M.C., Pavlovic S. (2014). Maritime route of colonization of Europe. Proc. Natl. Acad. Sci. USA.

[57] Fanis P., Skordis N., Frangos S., Christopoulos G., Spanou-Aristidou E., Andreou E., Manoli P., Mavrommatis M., Nicolaou S., Kleanthous M. (2018). Multiple endocrine neoplasia 2 in Cyprus: Evidence for a founder effect. J. Endocrinol. Investig..

[58] Neocleous V., Skordis N., Portides G., Efstathiou E., Costi C., Ioannou N., Pantzaris M., Anastasiadou V., Deltas C., Phylactou L.A. (2011). RET proto-oncogene mutations are restricted to codon 618 in Cypriot families with multiple endocrine neoplasia 2. J. Endocrinol. Investig..

[59] Al-Kurd A., Gross D.J., Zangen D., Atlan K., Mazeh H., Grozinsky-Glasberg S. (2018). Bilateral Medullary Thyroid Carcinoma in a 3-Year-Old Female Patient with Multiple Endocrine Neoplasia 2A Syndrome Undergoing Prophylactic Thyroidectomy: Should Current Guidelines Be Revised?. Eur. Thyroid J..

[60] Grozinsky-Glasberg S., Benbassat C.A., Tsvetov G., Feinmesser R., Peretz H., Shimon I., Lapidot M. (2007). Medullary thyroid cancer: A retrospective analysis of a cohort treated at a single tertiary care center between 1970 and 2005. Thyroid.

[61] Rosenblum Chava R., Hirsch D., Glasberg S., Benbassat C., Yoel U., Ishay A., Zolotov Lamprecht S., Bachar G., Banne E., Twito O. Clinical correlates of a large Israeli cohort of Cys 618 Arg RET mutation. Proceedings of the European Congress of Endocrinology 2022.

[62] Peretz H., Luboshitsky R., Baron E., Biton A., Gershoni R., Usher S., Grynberg E., Yakobson E., Graff E., Lapidot M. (1997). Cys 618 Arg mutation in the RET proto-oncogene associated with familial medullary thyroid carcinoma and maternally transmitted Hirschsprung’s disease suggesting a role for imprinting. Hum. Mutat..

[63] Machens A., Lorenz K., Weber F., Dralle H. (2018). Geographic epidemiology of medullary thyroid cancer families: Unearthing European ancestral heritage. Endocr. Relat. Cancer.

[64] Toledo R.A., Hatakana R., Lourenco D.M., Lindsey S.C., Camacho C.P., Almeida M., Lima J.V., Sekiya T., Garralda E., Naslavsky M.S. (2015). Comprehensive assessment of the disputed RET Y791F variant shows no association with medullary thyroid carcinoma susceptibility. Endocr. Relat. Cancer.

[65] Valente F.O., Dias da Silva M.R., Camacho C.P., Kunii I.S., Bastos A.U., da Fonseca C.C., Simiao H.P., Tamanaha R., Maciel R.M., Cerutti J.M. (2013). Comprehensive analysis of RET gene should be performed in patients with multiple endocrine neoplasia type 2 (MEN 2) syndrome and no apparent genotype-phenotype correlation: An appraisal of p.Y791F and p.C634Y RET mutations in five unrelated Brazilian families. J. Endocrinol. Investig..

[66] Aydogan B.I., Yuksel B., Tuna M.M., Navdar Basaran M., Akkurt Kocaeli A., Ertorer M.E., Aydin K., Guldiken S., Simsek Y., Cihan Karaca Z. (2016). Distribution of RET Mutations and Evaluation of Treatment Approaches in Hereditary Medullary Thyroid Carcinoma in Turkey. J. Clin. Res. Pediatr. Endocrinol..

[67] Larsen L.V., Mirebeau-Prunier D., Imai T., Alvarez-Escola C., Hasse-Lazar K., Censi S., Castroneves L.A., Sakurai A., Kihara M., Horiuchi K. (2020). Primary hyperparathyroidism as first manifestation in multiple endocrine neoplasia type 2A: An international multicenter study. Endocr. Connect..

[68] Basaran M.N., Tuna M.M., Karakilic E., Dogan B.A., Imga N.N., Berker D., Guler S. (2015). Characterization of V804M-mutated RET proto-oncogene associated with familial medullary thyroid cancer, report of the largest Turkish family. J. Endocrinol. Investig..

[69] Bergant D., Hocevar M., Besic N., Glavac D., Korosec B., Caserman S. (2006). Hereditary medullary thyroid cancer in Slovenia—Genotype-phenotype correlations. Wien. Klin. Wochenschr..

[70] Zupan A., Glavac D. (2015). The development of rapid and accurate screening test for RET hotspot somatic and germline mutations in MEN2 syndromes. Exp. Mol. Pathol..

[71] Zadnik V., Primic Zakelj M., Lokar K., Jarm K., Ivanus U., Zagar T. (2017). Cancer burden in slovenia with the time trends analysis. Radiol. Oncol..

[72] Milicevic S., Bergant D., Zagar T., Peric B. (2021). Crude annual incidence rate of medullary thyroid cancer and RET mutation frequency. Croat. Med. J..

[73] Katalinic D., Solter M., Nikolac N. RET M918T-exon 16 mutation in subjects with sporadic medullary thyroid cancer (sMTC). Proceedings of the 17th European Congress of Endocrinology.

[74] Abdelhakim A., Barlier A., Kebbou M., Benabdeljalil N., Timinouni M., Taoufiq F., Roche C., El Antri S. (2009). RET genetic screening in patients with medullary thyroid cancer: The Moroccan experience. J. Cancer Res. Ther..

[75] Efared B., Atsame-Ebang G., Tahirou S., Mazaz K., Hammas N., El Fatemi H., Chbani L. (2017). Bilateral pheochromocytoma with ganglioneuroma component associated with multiple neuroendocrine neoplasia type 2A: A case report. J. Med. Case Rep..

[76] Castinetti F., Waguespack S.G., Machens A., Uchino S., Hasse-Lazar K., Sanso G., Else T., Dvorakova S., Qi X.P., Elisei R. (2019). Natural history, treatment, and long-term follow up of patients with multiple endocrine neoplasia type 2B: An international, multicentre, retrospective study. Lancet Diabetes Endocrinol..

